# Extended-release calcifediol identifies a therapeutic vitamin D range in chronic kidney disease stages 3 and 4

**DOI:** 10.1093/ckj/sfag194

**Published:** 2026-06-10

**Authors:** John Choe, Akhtar Ashfaq, Charles W Bishop

**Affiliations:** OPKO Health, Inc., Miami, FL, USA; OPKO Health, Inc., Miami, FL, USA; OPKO Health, Inc., Miami, FL, USA

**Keywords:** 25-hydroxyvitamin D, chronic kidney disease, extended-release calcifediol, secondary hyperparathyroidism, vitamin D metabolism

## Abstract

**Background:**

Secondary hyperparathyroidism (SHPT) is a common complication of chronic kidney disease (CKD) driven by dysregulated vitamin D metabolism. The serum 25-hydroxyvitamin D (25D) levels required for targeted intact parathyroid hormone (iPTH) reductions are poorly defined, and conventional sufficiency thresholds of 20 or 30 ng/ml are inadequate. Exposure–response (E–R) modeling was used to define a 25D range associated with a 30% iPTH reduction during treatment with extended-release calcifediol (ERC).

**Methods:**

E–R relationships between 25D and iPTH were evaluated using pooled data from randomized clinical trials of ERC in participants with SHPT, CKD stage 3–4, and vitamin D insufficiency (VDI). Mixed-effects models characterized percent change in iPTH as a function of concurrent 25D. Mechanistic analyses evaluated associations between 25D, its key metabolites, and iPTH. Associations between achieved 25D and markers of mineral metabolism were assessed.

**Results:**

E–R modeling demonstrated a nonlinear relationship between 25D and iPTH reduction with a population-average of 84 ng/ml associated with 30% iPTH reduction (ER_30_), ranging from 67 to 103 ng/ml across the range of kidney function. Baseline body weight and sex significantly influenced achieved 25D concentrations. Higher achieved 25D was not associated with adverse changes in calcium, phosphorus, or fibroblast growth factor 23. Circulating 1,25-dihydroxyvitamin D was not independently associated with iPTH response.

**Conclusions:**

Elevation of 25D to 84 ng/ml (range: 66.9–102.6 ng/ml) during ERC treatment was associated with a 30% iPTH reduction in participants with SHPT, CKD stage 3–4, and VDI. This range was unassociated with adverse changes in mineral metabolism.

## Graphical Abstract

**Graphical Abstract:**
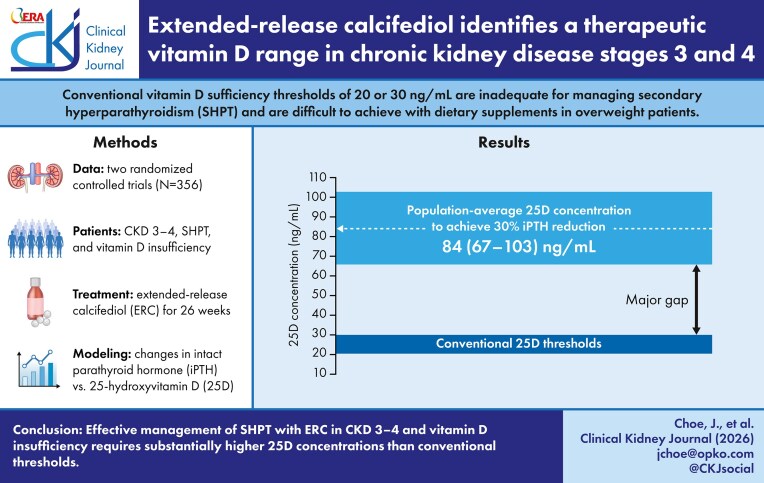

KEY LEARNING POINTS **What was known:**

- Serum 25-hydroxyvitamin D (25D) concentrations required for clinically meaningful (≥30%) reduction of elevated intact parathyroid hormone (iPTH) in chronic kidney disease (CKD) are poorly defined.
- Conventional vitamin D sufficiency thresholds of 20 or 30 ng/ml are inadequate for managing secondary hyperparathyroidism (SHPT) and can be difficult to achieve with dietary vitamin D supplements (cholecalciferol or ergocalciferol) in overweight CKD patients.
- Higher serum 25D concentrations (≥50 ng/ml) can drive sufficient extra-renal production of 1,25-dihydroxyvitamin D (1,25D) to correct dysregulated vitamin D metabolism arising from declining kidney function.

**This study adds:**

- Exposure–response modeling identified a population-average serum 25D concentration of 84 ng/ml (range of 67–103 ng/ml) associated with a 30% iPTH reduction (ER_30_) in patients with SHPT, CKD stage 3 or 4 and vitamin D insufficiency (VDI) treated with extended-release calcifediol (ERC).
- Circulating 25D, but not serum 1,25D, was independently associated with iPTH reduction, and body weight and sex influenced achieved 25D concentrations.
- Safety analyses found no associations between 25D concentration and serum calcium, phosphorus or fibroblast growth factor-23.

**Potential impact:**

- Effective management of SHPT in CKD stage 3 or 4 with ERC requires substantially higher serum 25D exposures than conventional vitamin D sufficiency thresholds of 20 or 30 ng/ml which cannot be reliably attained with dietary supplements (cholecalciferol or ergocalciferol) due to adiposity.
- Substantial inter-individual variability in iPTH response during ERC treatment supports a shift in the SHPT management paradigm from mere correction of VDI with fixed-dose approaches to individualized, exposure-based, response-anchored treatment regimens.
- Future studies will be required to define the ER_30_ for SHPT in other stages of CKD.

## INTRODUCTION

Chronic kidney disease (CKD) has a rising prevalence affecting 10%–14% of the global population [1]. A common and clinically important complication is secondary hyperparathyroidism (SHPT), driven by dysregulated vitamin D metabolism, characterized by elevated parathyroid hormone (PTH) concentrations and associated with mineral and bone disorder (MBD) and adverse cardiovascular outcomes [2, 3]. As kidney function declines, renal cytochrome P-450 25D-1α-hydroxylase (CYP27B1) is progressively impaired, limiting the conversion of 25-hydroxyvitamin D (25D) to its active hormone, 1,25-dihydroxyvitamin D (1,25D) [4]. Persistent vitamin D insufficiency (VDI) further limits substrate availability for 1,25D production, contributing to inadequate activation of the vitamin D receptor (VDR) in the parathyroid glands and resulting in excessive PTH secretion [2, 3, 5].

Reduced serum 1,25D exposure has an early onset in CKD and becomes more pronounced as the disease progresses [6]. A central therapeutic objective is to restore adequate 1,25D–VDR signaling in the parathyroid glands [7]. The usual treatment option is oral calcitriol or a 1α-hydroxylated analogue, either of which effectively suppresses elevated PTH. Such hormone replacement therapy directly increases active intestinal absorption of dietary calcium and phosphorus, raising the risk of hypercalcemia, hyperphosphatemia, and vascular calcification [7, 8]. Accordingly, the current Kidney Disease Improving Global Outcomes (KDIGO) clinical practice guideline suggests against its routine use in non-dialysis CKD [9].

An alternative treatment option derives from evidence that 1,25D can be produced outside the kidney when sufficient substrate (25D) is made available [10–12]. Extra-renal CYP27B1 is broadly expressed beyond the kidney, including in the parathyroid glands, where it enables local production of 1,25D in proportion to serum 25D concentrations [13–15]. Therapeutic elevation of serum 25D with extended-release calcifediol (ERC) can enable extra-renal 1,25D production when kidney function is reduced [16, 17]. Under this substrate-driven framework, serum 25D functions as the primary determinant of tissue-level vitamin D signaling activity.

Delivering sufficient 25D to extra-renal CYP27B1 can be challenging with dietary vitamin D supplements (cholecalciferol or ergocalciferol), especially in overweight CKD patients [18–21]. These supplements are highly lipophilic and accumulate in adipose tissue, from which they are poorly mobilized due to low affinity for the circulating vitamin D-binding protein (DBP) [21, 22]. Further, hepatic 25-hydroxylation of these supplements is impaired in overweight CKD patients [23], limiting the intended elevation of serum 25D. Calcifediol (25D_3_) exhibits more favorable pharmacodynamics owing to its greater polarity, higher DBP binding affinity, minimal adipose sequestration, and lack of dependency on hepatic activation [21, 24, 25]. Importantly, calcifediol does not avidly bind VDR and cannot directly stimulate intestinal calcium and phosphorus absorption, differentiating it from calcitriol and its analogues [17].

Calcifediol in an extended-release formulation (ERC) produces a gradual rise in serum 25D levels, avoiding abrupt elevations that can overwhelm mineral and hormonal regulatory pathways, thereby providing an improved therapeutic profile over immediate-release calcifediol [26–28]. Multiple randomized clinical trials (RCTs) and real-world studies have demonstrated that ERC safely and sufficiently raises serum 25D concentrations in overweight CKD patients, increasing serum 1,25D, and reducing elevated intact PTH (iPTH) despite declining kidney function [16, 29–31]. Importantly, sustained and clinically meaningful iPTH reductions (≥30%) achieved with ERC in patients with CKD stage 3 or 4 have been associated with slower decline of estimated glomerular filtration rate (eGFR) [28]. Such reductions required mean 25D concentrations of ∼89 ng/ml, highlighting the need for higher-than-conventional 25D exposures in this population.

Serum 25D concentrations needed for PTH reduction of ≥30% remain poorly defined in patients with CKD stage 3 or 4. The current KDIGO guideline does not specify a therapeutic target for serum 25D in CKD [9]. Consequently, vitamin D repletion therapy has been anchored to sufficiency thresholds intended for the general population rather than guided by 25D exposure and biochemical response [9, 32]. This gap underscores the need for a quantitative framework linking achieved 25D concentrations to iPTH reduction.

In view of this gap, the analysis described herein applied exposure–response (E–R) modeling to characterize the relationship between achieved 25D concentration and iPTH reduction in patients with SHPT, CKD stage 3 or 4, and VDI. Additional analyses examining the relationship between 25D and its metabolites to iPTH, and determinants of achieved 25D exposure, were conducted to support interpretation of the E–R finding. Using a population-average framework, E–R modeling identified a clinically relevant 25D range associated with 30% iPTH reduction which accounted for variability in kidney function. The safety of 25D concentrations within this exposure range was assessed using markers of mineral metabolism.

## MATERIALS AND METHODS

### Study design and population

This post-hoc analysis used pooled data from two identical randomized, double-blind, placebo-controlled clinical trials (NCT1651000, NCT01704079) evaluating 26 weeks of daily oral ERC treatment in participants with SHPT, CKD stage 3 or 4, and VDI, operationally defined as serum total 25D < 30 ng/ml. Trial design and primary outcomes have been published previously [30].

The analysis included 356 participants randomized to ERC (*n* = 234) or placebo (*n* = 122) who completed the studies per protocol. ERC dosing was initiated at 30 µg, with protocol-defined up-titration to 60 µg based on biochemical response after 12 weeks of treatment. Both studies were conducted in accordance with the Declaration of Helsinki, with institutional review board approval and written informed consent from all participants.

### Baseline characteristics

Baseline characteristics were summarized as mean (SD) for continuous variables and number of subjects (%) for categorical variables. Continuous variables were compared using Wilcoxon rank-sum tests, and categorical variables were compared using chi-square or Fisher’s exact tests, as appropriate. Baseline values were defined as the mean of post-washout pre-dose assessments, and Week 12 (mid-study) and Week 26 (end of study) values were defined as the protocol-specified averages of assessments during Weeks 8–12 and Weeks 20–26, respectively, to reduce short-term variability and assay imprecision.

### Achieved 25-hydroxyvitamin D exposure

Serum 25D concentrations were analyzed over the 26-week treatment period using linear mixed-effects models with fixed effects for treatment week, titration status, and their interaction, and participant-specific random effects. Models were adjusted for prespecified baseline covariates, including age, sex, race, body weight, eGFR, iPTH, serum 25D, visit timing, and seasonality (using sine and cosine terms). Concomitant low-level vitamin D supplementation was included in models of achieved serum 25D but excluded from E–R analyses in which achieved serum 25D served as the primary explanatory variable.

### Analyses of 25-hydroxyvitamin D, its metabolites and intact parathyroid hormone

Adjusted least-squares means for 25D, 1,25D, 24,25-dihydroxyvitamin D (24,25D), and iPTH were estimated using mixed-effects models with fixed effects for treatment week, CKD stage, and their interaction, and a random intercept for participant. Estimates (95% CIs) were obtained within CKD stage. Week 26 changes from baseline were derived using model-based contrasts, and baseline comparisons between CKD stages 3 and 4 were performed using linear models.

Mechanistic analyses were conducted to evaluate relationships among 25D and its key metabolites and iPTH. Longitudinal changes in serum 25D, 1,25D, 24,25D, and iPTH were assessed using mixed-effects models, from which adjusted least-squares means (LS means) and 95% CIs were estimated at each visit within CKD stage. Associations among these parameters were evaluated using Pearson correlation analyses at Weeks 12 and 26. Multivariable mixed-effects models were further used to assess the independent contributions of achieved 25D and circulating 1,25D to iPTH levels, adjusting for baseline covariates.

### Exposure–response modeling

The E–R relationship between achieved serum 25D and iPTH was evaluated by modeling percent reduction in iPTH as a continuous function of concurrent serum 25D using spline-based mixed-effects models adjusted for baseline covariates. Percent reduction was selected to account for inter-individual variability in baseline iPTH and to enable clinically interpretable comparisons across participants with differing disease severity. Data from all participants (treated with ERC or placebo) were included to capture the full observed 25D exposure range. Post-baseline observations at Weeks 12 and 26 were modeled jointly, with Week 26 predictions used for clinical interpretation.

Within this framework, population-average serum 25D concentrations associated with specified levels of iPTH reduction were estimated by marginalizing over the observed distribution of baseline covariates. In particular, the 25D concentration associated with a predicted 30% reduction in iPTH (ER_30_) was evaluated as a primary reference point, consistent with the widely accepted regulatory efficacy endpoint for SHPT therapies (e.g. ERC, paricalcitol, doxercalciferol, cinacalcet, etelcalcetide). Corresponding 25D concentrations associated with 10% and 20% reductions in iPTH (ER_10_ and ER_20_) were also estimated to support clinically relevant interpretation across a range of treatment responses.

A clinically relevant 25D range was defined around each reference point (e.g. ER_30_) by accounting for variability across kidney function. Specifically, 25D concentrations associated with a 30% reduction in iPTH (ER_30_) were estimated at the 5th and 95th percentiles of eGFR corresponding to 17 and 50 ml/min/1.73 m^2^, respectively, while marginalizing over all remaining covariates. CIs were derived from simulations based on the fixed-effect covariance matrix. Sensitivity analyses evaluated the robustness of the E–R relationship to inclusion of additional covariates, including baseline calcium.

### Safety analyses

Associations between achieved serum 25D and safety markers (serum calcium, phosphorus, and FGF23) were evaluated using longitudinal mixed-effects models. To distinguish within- and between-individual effects, serum 25D was decomposed into participant-specific mean values and within-individual deviations. Joint Wald tests were used to assess within-individual associations.

### Bioanalytical methods and software

Blood samples were analyzed at central laboratories using methods validated per the stringent requirements of the United States Food and Drug Administration. Serum total 25D and 24,25D were measured by liquid chromatography tandem mass spectrometry (LC–MS/MS), serum total 1,25D by chemiluminescence (Diasorin Liaison), and plasma iPTH by electrochemiluminescence (Roche Elecsys Cobas).

All analyses were performed using R version 4.5.2. Two-sided *P*-values < .05 were considered statistically significant. 

## RESULTS

### Population characteristics

Baseline characteristics of 234 participants who completed 26 weeks of ERC treatment per protocol are summarized in Table 1. Of these, 194 (83%) were up-titrated to 60 µg at the beginning of Week 13. Up-titrated participants were younger, more frequently male, less frequently Hispanic, and had higher body weight, lower serum 1,25D and calcium concentrations, and higher iPTH levels. Race distribution, body mass index (BMI), total 25D, phosphorus, FGF23, and eGFR were similar between the up-titrated group and those who remained at 30 µg.

**Table 1:** Baseline characteristics.

Parameter, unit (% or SD)	All active participants (*N* = 234)	Non-titrated (*N* = 40)	Up-titrated (*N* = 194)	*P*-value^a^
Age, years	66.0 (10.42)	69.4 (8.48)	65.3 (10.67)	.035
Sex, *n*				.043
Female	118 (50.4)	26 (65.0)	92 (47.4)	
Male	116 (49.6)	14 (35.0)	102 (52.6)	
Race, *n*				.825
Asian	3 (1.3)	0	3 (1.5)	
Black	148 (63.2)	28 (70.0)	120 (61.9)	
White	79 (33.8)	12 (30.0)	67 (34.5)	
Other	4 (1.7)	0	4 (2.1)	
Hispanic or Latino, *n*	44 (18.8)	14 (35.0)	30 (15.5)	.004
Weight, kg	98.4 (25.48)	90.7 (24.53)	100.0 (25.44)	.037
BMI, kg/m^2^	34.7 (8.17)	33.9 (8.63)	35.0 (8.08)	.452
Total 25D, ng/ml	19.7 (5.34)	21.1 (5.66)	19.4 (5.24)	.076
1,25D, pg/ml	34.4 (13.23)	39.5 (13.45)	33.4 (12.98)	.006
24,25D, ng/ml	1.0 (0.4)	1.1 (0.4)	1.0 (0.4)	.724
iPTH, pg/ml	143.7 (57.31)	127.9 (52.27)	146.9 (57.89)	.004
Calcium, mg/dl	9.2 (0.30)	9.4 (0.35)	9.2 (0.27)	<.001
Phosphorus, mg/dl	3.8 (0.56)	3.7 (0.46)	3.8 (0.57)	.235
FGF23, pg/ml	41.4 (43.23)	38.7 (27.74)	41.8 (45.33)	.781
eGFR, ml/min/1.73 m^2^	30.6 (10.22)	31.6 (9.32)	30.3 (10.41)	.331

Note: BMI, body mass index; 25D, 25-hydroxyvitamin D; 1,25D, 1,25-dihydroxyvitamin D; 24,25D, 24,25-dihydroxyvitamin D; iPTH, intact parathyroid hormone; FGF23, fibroblast growth factor 23; eGFR, estimated glomerular filtration rate.

a *P*-values are from Wilcoxon rank-sum tests for continuous variables and chi-square or Fisher’s exact tests, as appropriate, for categorical variables and reflect unadjusted distributional differences between groups.

### Serum 25-hydroxyvitamin D exposure

**Figure 1:**
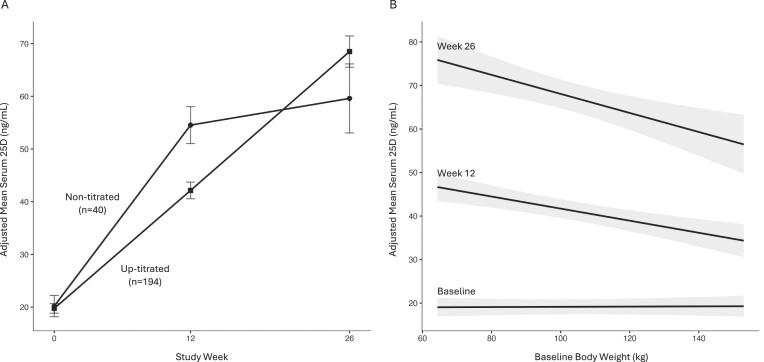
Achieved serum 25-hydroxyvitamin D (25D) concentrations by dose titration status and baseline body weight during 26 weeks of treatment with extended-release calcifediol (ERC). (A) Adjusted mean serum 25D concentrations over time in non-titrated or up-titrated participants. Points represent adjusted means and error bars indicate 95% CIs. (B) Adjusted mean serum 25D concentrations as a function of baseline body weight among up-titrated participants (*n* = 194). Shaded bands denote 95% CIs.

Adjusted mean serum 25D concentrations by titration status are shown in Fig. 1A. At baseline, adjusted mean (95% CI) concentrations were similar between groups [20.2 (18.2–22.2) vs. 19.8 (18.8–20.7) ng/ml]. By Week 12, serum 25D had increased in both groups, with lower concentrations observed in the group that was up-titrated at Week 13 [42.1 (40.5–43.7) vs. 54.5 (51.0–58.0) ng/ml; *P* < .001). By Week 26, mean 25D had increased to 68.5 (65.5–71.5) ng/ml in the up-titrated group (*P* = .0156), whereas the non-titrated group showed only a nominal additional increase to 59.6 (53.0–66.1) ng/ml.

Mean achieved 25D was inversely associated with baseline body weight (Fig. 1B). At Week 12, each 10-kg decrease in body weight corresponded to a mean increase of 1.4 (0.8–2.0; *P* < .001) ng/ml in achieved 25D concentration. At Week 26, the association became a mean 2.2 (1.0–3.4; *P* = 0.003) ng/mL increase per 10 kg decrease. Participants with lower body weight achieved higher 25D concentrations at either ERC dose (30 or 60 µg). Body weight, rather than BMI, was a more informative determinant of inter-individual variability in 25D exposure. The observed inverse relationship was similar for both sexes; however, women achieved higher 25D concentrations at Week 12 [43.9 (41.2–46.7) vs. 39.6 (37.0–42.2) ng/ml; *P* = .006] and Week 26 [73.0 (68.5–77.5) vs. 63.5 (59.2–67.8) ng/ml; *P* = .002] despite similar baseline concentrations [19.2 (17.1–21.2) vs. 19.1 (17.3–20.9) ng/ml; *P* = .94].

### Associations between serum 25-hydroxyvitamin D and its key metabolites

Increases in serum 25D were accompanied by increases in serum 1,25D and 24,25D (Table 2). Concentrations of both metabolites were consistently higher in participants with stage 3 CKD. Circulating 24,25D levels were moderately correlated with achieved serum 25D concentrations at baseline (*r* = 0.47; *P* < .001) and strongly correlated during treatment at Week 12 and Week 26 (both *r* = 0.71; *P* < .001). In contrast, circulating 1,25D levels were less strongly correlated with 25D concentrations across the entire treatment period, with correlation coefficients ranging from 0.11 to 0.16.

**Table 2:** Adjusted least-squares means of vitamin D metabolites and iPTH by treatment duration and CKD stage (ERC-treated participants).

Analyte	CKD Stage	Baseline	Week 12	Week 26	Week 26 Change from Baseline
25D, ng/ml	All	19.7 (17.7, 21.7)	44.2 (42.2, 46.2)^c^	67.1 (65.1, 69.0)^c^	47.4 (45.3, 49.5)
	Stage 3	20.3 (17.6, 23.0)	45.1 (42.4, 47.8)^c^	66.5 (63.8, 69.2)^c^	46.3 (43.3, 49.2)
	Stage 4	19.2 (16.3, 22.0)	43.3 (40.5, 46.1)^c^	67.7 (64.8, 70.5)^c^	48.5 (45.4, 51.6)
1,25D, pg/ml	All	34.2 (32.4, 35.9)	43.2 (41.5, 45.0)^c^	46.3 (44.5, 48.0)^c^	12.1 (10.6, 13.7)
	Stage 3	39.2 (36.7, 41.6)	49.9 (47.5, 52.4)^c^	53.9 (51.5, 56.4)^c^	14.7 (12.6, 16.9)
	Stage 4	29.2 (26.6, 31.7)^c^	36.6 (34.0, 39.1)^c^	38.6 (36.1, 41.2)^c^	9.5 (7.2, 11.7)^a^
24,25D, ng/ml	All	0.9 (0.6, 1.1)	2.4 (2.1, 2.6)^c^	3.8 (3.6, 4.0)^c^	3.0 (2.7, 3.2)
	Stage 3	1.0 (0.7, 1.2)	2.6 (2.3, 2.9)^c^	4.3 (4.0, 4.5)^c^	3.3 (3.0, 3.6)
	Stage 4	0.8 (0.4, 1.1)^a^	2.1 (1.8, 2.4)^c^	3.4 (3.1, 3.7)^c^	2.6 (2.3, 2.9)^b^
iPTH, pg/ml	All	144.6 (137.0, 152.2)	125.5 (118.0, 133.1)^c^	112.5 (105.0, 120.1)^c^	-32.1 (-37.1, -27.0)
	Stage 3	125.9 (115.4, 136.3)	107.1 (96.7, 117.6)^c^	97.3 (86.9, 107.8)^c^	-28.6 (-35.5, -21.6)
	Stage 4	163.3 (152.3, 174.3)^c^	144.0 (133.0, 155.0)^c^	127.7 (116.7, 138.7)^c^	-35.6 (-42.9, -28.3)

Note: Values are adjusted least-squares means (95% CIs). Visit-specific estimates were obtained from mixed-effects models including visit and CKD stage, with subject as a random effect, using ERC-treated participants only. 25D, 25-hydroxyvitamin D; 1,25D, 1,25-dihydroxyvitamin D; 24,25D, 24,25-dihydroxyvitamin D; iPTH, intact parathyroid hormone; ERC, extended-release calcifediol; CKD, chronic kidney disease.

*P*-values for Week 12 and Week 26 indicate model-based comparisons vs. baseline within each CKD stage.

Week 26 change from baseline column shows model-based estimates of change; superscripts denote comparison between CKD stage 3 and stage 4 for change from baseline at Week 26.

Baseline stage 3 vs. stage 4 comparisons were obtained from baseline-only linear models; superscripts denote comparisons between CKD stage 3 and stage 4 at baseline.

a *P* < .05.

b *P* < .01.

c *P* < .001. All *P*-values are nominal (unadjusted for multiplicity).

### Serum 25-hydroxyvitamin D and 1,25-dihydroxyvitamin D versus intact parathyroid hormone

Serum 25D concentrations showed modest but consistent inverse correlations with iPTH levels at baseline (*r* = −0.16; *P* = .05), Week 12 (*r* = −0.22; *P* = .01), and Week 26 (*r* = −0.24; *P* < .001), with the strength of association increasing over time. In contrast, serum 1,25D concentrations were not correlated with iPTH levels at baseline (*r* = −0.05; *P* = .49), Week 12 (*r* = −0.02; *P* = .75), or Week 26 (*r* = −0.02; *P* = .74), with no observable relationship across the range of concentrations. Multivariable analysis demonstrated that higher achieved serum 25D, but not 1,25D, concentrations were independently and inversely associated with lower iPTH levels.

### Exposure–response relationship: 25-hydroxyvitamin D versus intact parathyroid hormone

**Figure 2:**
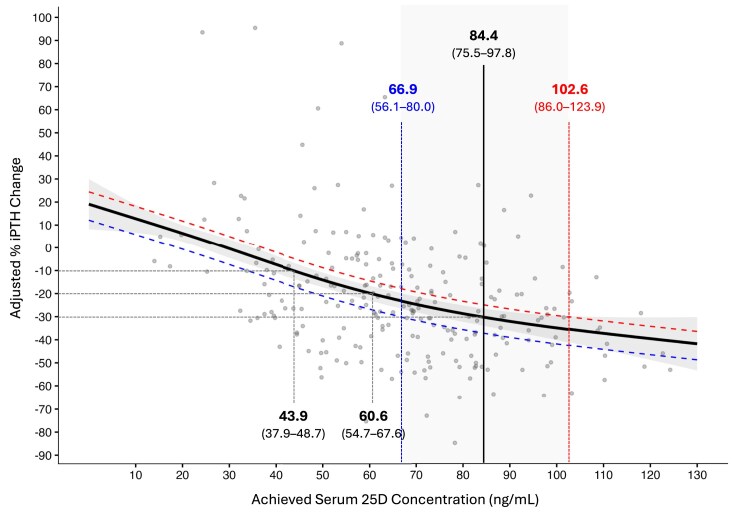
Population-average exposure–response relationships between achieved serum 25-hydroxyvitamin D (25D) and % change in intact parathyroid hormone (iPTH). The solid black curve represents the marginal mean response, and the shaded band indicates the 95% CI. The solid vertical line indicates population-average 25D concentration associated with a 30% iPTH reduction. Dashed curves represent population-average estimates with estimated glomerular filtration rate (eGFR) fixed at the 5th percentile (red) and the 95th percentile (blue) and vertical dashed lines indicate the corresponding 25D concentrations associated with a 30% reduction in iPTH under each scenario. Vertical grey dashed lines indicate population-average 25D concentrations associated with 10% and 20% reductions in iPTH.

E–R modeling showed that achieved serum 25D concentration was the primary determinant of percent iPTH reduction (*P* < .001). The relationship was nonlinear and monotonic, with progressively greater reductions in iPTH observed at higher 25D concentrations (Fig. 2). The population-average ER_30_ was 84.4 (75.5–97.8) ng/ml.

ER_30_ concentrations after fixing eGFR at the 95th and 5th percentiles were 66.9 (56.1–80.0) and 102.6 (86.0–123.9) ng/ml, respectively, defining a clinically relevant 25D range of 66.9–102.6 ng/ml. Population-average 25D concentrations corresponding to 10% and 20% reductions in iPTH were estimated at 43.9 (37.9–48.7) and 60.6 (54.7–67.6) ng/ml, respectively.

Among baseline covariates, eGFR was the only significant independent predictor of percent iPTH reduction (β ≈ −0.37% per ml/min/1.73 m²; *P* = .0017). In a sensitivity analysis, baseline serum calcium was associated with a numerically, but not significantly, greater reduction in iPTH (β ≈ −5.39; *P* = .10) after adjustment for achieved 25D and other covariates. Importantly, inclusion of baseline calcium in the model did not materially alter the E–R relationship between achieved 25D and iPTH reduction.

### Associations between 25-hydroxyvitamin D and safety parameters

**Figure 3:**
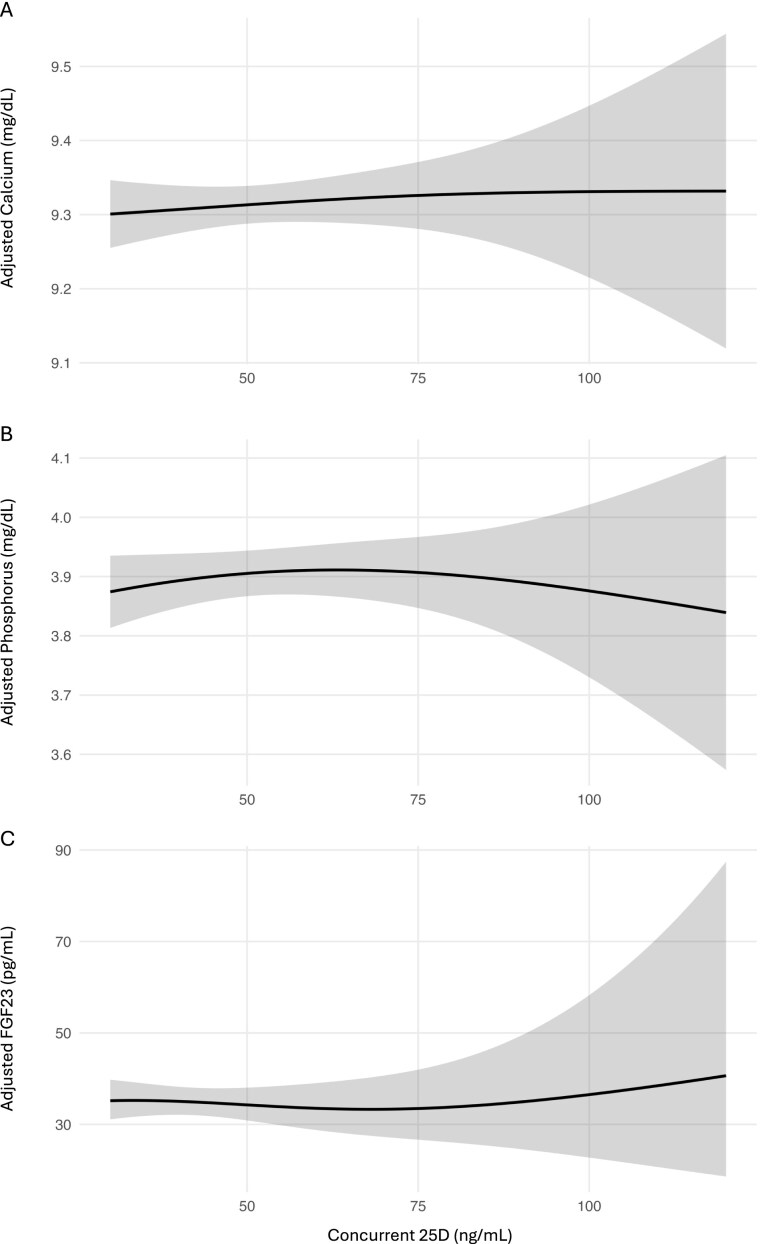
Population-average adjusted relationships between achieved serum 25-hydroxyvitamin D (25D) concentrations and serum (A) calcium, (B) phosphorus, and (C) fibroblast growth factor 23 (FGF23). Curves represent marginal mean predictions from longitudinal mixed-effects models; shaded bands denote 95% CIs.

Longitudinal analyses showed no associations between achieved serum 25D concentrations and increases in serum calcium, phosphorus, and FGF23 (Fig. 3). Population-average prediction curves demonstrated stability in these safety markers across the observed 25D range of ∼30–120 ng/ml. Joint Wald tests of within-individual changes in 25D confirmed no significant association with calcium (*P* = .91), phosphorus (*P* = .53), and FGF23 (*P* = .34). Across models, these parameters were primarily determined by baseline values and renal function, rather than achieved 25D concentrations. The population-average ER_30_ (66.9–102.6 ng/ml) fell within this observed safety window.

Confirmed episodes of hypercalcemia (2 consecutive visits with serum calcium >10.3 mg/dl) were infrequent during the study, occurring in 6 (2.1%) out of 285 participants in the safety population [30]. Episodes were not associated with dose, serum 25D or 1,25D concentrations, or with use of elemental calcium or phosphate binder therapy. Notably, five of the six participants had a predisposition to elevated serum calcium, with values ≥9.8 mg/dl during the screening period.

## DISCUSSION

This post-hoc analysis applied E–R modeling to pooled data from two RCTs conducted with ERC in patients with SHPT, stage 3 or 4 CKD and VDI. The modeling demonstrated a nonlinear, monotonic relationship between serum 25D and iPTH-lowering response and identified a population-average 25D concentration of 84.4 ng/ml (range: 66.9–102.6) associated with a predicted 30% iPTH reduction. Reduction of 30% is a widely accepted regulatory efficacy endpoint for SHPT therapies and was the primary efficacy end point of the relevant ERC trials. Notably, the ER_30_ and associated range identified for ERC exceeded 25D sufficiency thresholds of 20 or 30 ng/ml derived for the general population [32, 33] yet remained unassociated with adverse changes in mineral metabolism.

Dose–exposure analysis in this patient population showed that ERC titration from 30 to 60 µg per day produced predictable increases in serum 25D concentrations with substantial inter-individual variation. Baseline body weight emerged as a key determinant of achieved 25D concentrations (consistent with differences in volume of distribution related to adiposity), as did sex (with women attaining higher concentrations at comparable body weight). These findings indicate that variability in 25D exposure under fixed dosing reflects multiple patient-specific factors beyond dose alone and support individualized dose titration with ERC to ensure effective SHPT treatment.

The observed iPTH-lowering responses were similar in the CKD stage 3 and 4 subgroups despite eGFR being a significant factor in the modeling. Higher baseline iPTH in stage 4 requires larger absolute change and thus higher 25D exposure for equivalent percent reductions. It follows that no single 25D concentration could have ensured consistent iPTH response across all levels of kidney function. The identified 25D range (66.9–102.6 ng/ml) for ER_30_ reflects variability in kidney function and should be interpreted as a response-anchored guide for individualized SHPT treatment without implying a universally applicable threshold. Lower 25D concentrations, namely 43.9 and 60.6 ng/ml, corresponded to more moderate reductions in iPTH of 10% and 20%, respectively.

Achieved 25D concentrations and iPTH-lowering responses were nearly identical in participants with stage 3 CKD versus those with stage 4 disease, despite markedly lower serum 1,25D concentrations in the latter subgroup. Notably, no significant association was observed between circulating 1,25D and percent change in iPTH, whereas serum 25D emerged as the primary independent predictor of iPTH reduction. Although circulating 1,25D increased in parallel with 25D, it did not independently associate with iPTH reduction. These findings are consistent with substrate-driven 1,25D production and local VDR activation within the parathyroid gland, whereby circulating 1,25D does not adequately reflect vitamin D signaling at the tissue level.

The E–R association between achieved 25D and iPTH reduction was robust to adjustment for baseline calcium and unaltered by other differences in pre-treatment mineral metabolism. No associations were observed between achieved 25D and increases in serum calcium, phosphorus, or FGF23 across the observed exposure range of ∼30–120 ng/ml. The 25D range of 66.9–102.6 ng/ml identified in this analysis lies well within this observed safety window, providing essential safety context for exposure-based treatment with ERC.

The current KDIGO guideline does not define evidence-based serum 25D thresholds for managing CKD-MBD [9], and clinical practice is often guided by conventional sufficiency thresholds derived from the general population [32, 33]. There often is reluctance to raise serum 25D above 50 ng/ml deriving largely from safety concerns expressed by the Institute of Medicine (now National Academy of Medicine) [31], which are not applicable to CKD patients. The present findings suggest that higher serum 25D exposures are required in CKD to support substrate-driven extra-renal 1,25D production and to compensate for declining hormone production in impaired kidneys [17].

To our knowledge, this is the first study to characterize a clinically relevant serum 25D range for treating CKD-related SHPT. Prior analyses based on categorical 25D concentration achieved with ERC and a binary responder definition have demonstrated that higher achieved concentrations (≥50 ng/ml) were associated with a greater likelihood of ≥30% iPTH reduction [16]. The present work extends those findings by modeling achieved 25D as a continuous exposure variable and defining clinically interpretable ranges associated with graded (10%, 20%, or 30%) iPTH reductions.

Several limitations should be acknowledged. This was a post-hoc analysis and, as such, should be considered hypothesis-generating. Dose titration was not randomized with respect to achieved 25D concentrations, and residual confounding may remain despite appropriate statistical adjustment. Joint modeling of post-baseline observations assumed a stable E–R relationship over time, which is supported by the known pharmacodynamic properties of vitamin D metabolism. Finally, generalizability may be limited to populations and treatment conditions similar to those studied.

In conclusion, elevation of 25D to ∼84 ng/ml (range: 66.9–102.6) during ERC treatment was associated with 30% iPTH reductions in participants with SHPT, CKD stage 3 or 4, and VDI. This exposure range substantially exceeded conventional 25D sufficiency thresholds derived for the general population yet remained unassociated with adverse changes in mineral metabolism. These findings support a shift from insufficiency-correction paradigms toward an individualized, exposure-based, and response-anchored approach to 25D repletion with ERC therapy and provide a quantitative framework for interpreting serum 25D concentrations in CKD-related SHPT.

## Data Availability

The data supporting the findings of this study are available from the corresponding author upon reasonable request, subject to applicable privacy restrictions.
